# Supplementary material for: Deep learning extends de novo protein modelling coverage of genomes using iteratively predicted structural constraints
Source: Nat Commun. 2019 Sep 4;10:3977. doi: 10.1038/s41467-019-11994-0 (PMC6726615; doi:10.1038/s41467-019-11994-0)
Supplement: Supplementary file 1 — Supplementary Information [file 41467_2019_11994_MOESM1_ESM.pdf]

Deep learning extends *de novo* protein modelling coverage of genomes using iteratively predicted structural constraints

Greener et al. 2019

Supplementary Information

Supplementary Figure 1 Run time of DMPfold on the CASP12 FM domains. Each of the 22 domains has the total DMPfold run time, the input generation time (mainly alignment generation and running PSICOV) and the model generation time (mainly running CNS). The total time is the sum of the input generation and model generation time. In each case a single Intel Xeon Processor E5-2640 v3 with 16 GB RAM was used. T0941-D1, indicated with an asterisk, is an outlier with input generation time 26 hours and total time 31 hours due to PSICOV taking a long time to converge. This value was omitted when calculating the line of best fit for the total times.

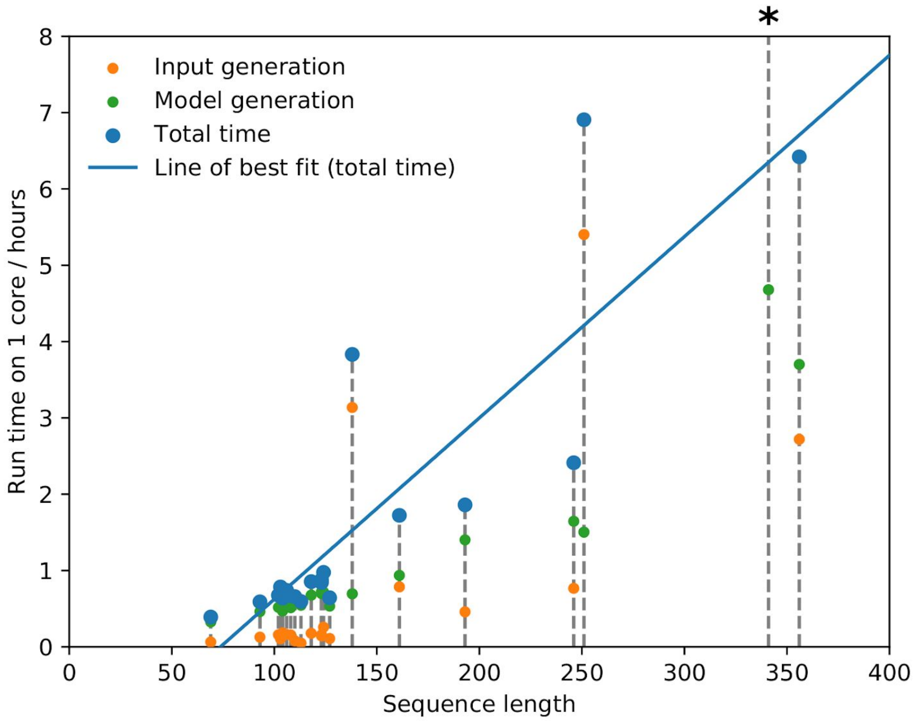

Supplementary Figure 2 Correlation between real and predicted TM-align scores on the Pfam validation set. Cross-validation was used and the predicted value reported when that protein was in the hold-out set. The Pearson correlation coefficient between the two sets is 0.733.

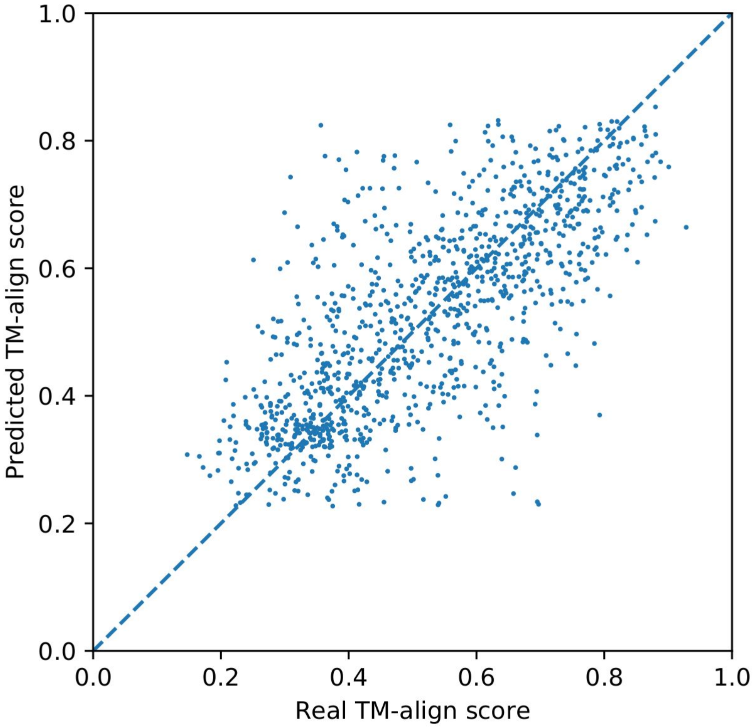

Supplementary Table 1 Ablation study on the CASP12 FM domains. In each case the results are reported when a certain combination of constraint types are used in the CNS step. The top model only is considered. The first row is the same as the first row in Table 1. The rows are ordered by descending mean TM-score.

**Distance constraints used**	**Torsion constraints used**	**H-bond constraints used**	**Mean TM-score**	**Median TM-score**	**TM-scores above 0.5**
✔	✔	✔	0.46	0.49	11
✔	✔	✘	0.44	0.45	10
✔	✘	✔	0.43	0.46	9
✔	✘	✘	0.42	0.39	8
✘	✔	✔	0.23	0.23	0
✘	✘	✔	0.18	0.17	0
✘	✔	✘	0.15	0.14	0
✘	✘	✘	0.11	0.11	0

Supplementary Table 2 Data for several model proteomes, showing the total numbers of residues in various proteomes that are covered by Pfam annotations, and the number of these that can either be covered by homology models or by DMPfold predictions.

TaxID	Species name	Total residues	Residues with Pfam annotations	Pfam-annotated residues with detectable templates	High-confidence DMPfold predictions	Lower-confidence DMPfold predictions
9606	*Homo sapiens*	19,822,311	9,983,581	8,900,364	138,951	944,266
3702	*Arabidopsis thaliana*	13,903,965	7,199,207	6,416,574	224,877	557,756
10116	*Rattus norvegicus*	14,582,536	7,448,281	6,795,191	86,831	566,259
6239	*Caenorhabditis elegans*	9,092,225	4,606,770	4,217,739	143,242	245,789
10090	*Mus musculus*	19,373,020	9,644,370	8,717,011	116,853	810,506
7955	*Danio rerio*	16,913,759	8,572,277	7,861,665	129,271	581,341
44689	*Dictyostelium discoideum*	4,425,280	1,895,003	1,721,926	47,269	125,808
8355	*Xenopus laevis*	16,097,238	7,886,407	7,204,047	124,656	557,704
7227	*Drosophila melanogaster*	12,412,460	5,053,389	4,667,547	79,785	306,057
4577	*Zea mays*	35,774,997	16,819,170	15,159,555	398,232	1,261,383
83333	*Escherichia coli* (strain K12)	1,292,975	973,800	902,804	37,580	33,416
559292	*Saccharomyces cerevisiae*	2,482,183	1,278,158	1,147,013	29,543	101,602
39946	*Oryza sativa* subsp. *indica*	10,614,423	5,391,613	4,773,665	204,349	413,599
36329	*Plasmodium falciparum*	2,252,325	866,889	811,228	15,132	40,529
	**Total**	179,039,697	87,618,915	79,296,329	1,776,571	6,546,015

Supplementary Table 3 Numbers of UniProt entries listed in the Pfam proteome assignments for several proteomes. Only active entries with at least one Pfam annotation were considered. The number of entries either gaining or not gaining new, high-confidence DMPfold models is shown for entries that either have or do not have (at least partial) coverage by direct PDB hits or templates.

TaxID	Species name	Total entries with PDB coverage	Total entries without PDB coverage	Have PDB coverage, no high-confidence DMPfold models	Have PDB coverage, additional coverage by DMPfold models	No PDB coverage, no high-confidence DMPfold models	No PDB coverage, high-confidence DMPfold models available
9606	*Homo sapiens*	42,371	5,051	42,175	196	4,261	790
3702	*Arabidopsis thaliana*	26,558	3,298	26,278	280	2,295	1,003
10116	*Rattus norvegicus*	23,882	2,331	23,746	136	1,950	381
6239	*Caenorhabditis elegans*	15,097	1,781	14,932	165	1,008	773
10090	*Mus musculus*	35,095	4,023	34,901	194	3,443	580
7955	*Danio rerio*	28,532	2,396	28,316	216	1,863	533
44689	*Dictyostelium discoideum*	6,411	780	6,373	38	567	213
8355	*Xenopus laevis*	26,369	2,367	26,140	229	1,875	492
7227	*Drosophila melanogaster*	15,137	1,579	15,042	95	1,106	473
4577	*Zea mays*	61,460	6,428	60,792	668	4,550	1,878
83333	*Escherichia coli* (strain K12)	3,588	395	3,547	41	210	185
559292	*Saccharomyces cerevisiae*	4,195	533	4,168	27	379	154
39946	*Oryza sativa* subsp. *indica* (Rice)	20,482	2,974	20,248	234	1,968	1,006
36329	*Plasmodium falciparum*	2,955	234	2,937	18	170	64
	**Total**	312,132	34,170	309,595	2,537	25,645	8,525

Supplementary Table 4 Structures for high-confidence modelled Pfam families released in 2019, i.e. after the PDB was searched for templates for this study. This set acts as a further validation set for DMPfold.

**Pfam ID**	**PDB ID**	**Chain ID**	**TM-align score of model**
PF00810	6I6H	A	0.64
PF02405	6IC4	G	0.70
PF03808	5WB4	A	0.69
PF04064	6HB1	A	0.62
PF06761	6IXH	P	0.24
PF08742	6N29	A	0.76
PF09856	6CYY	A	0.62
PF13839	6CCI	A	0.74
PF14473	6DRF	A	0.58
		**Mean**	0.62
		**Median**	0.64

Supplementary Table 5 Listing of all DMPfold input features and their contributions to the input feature tensor. For features defined on single residues, the feature values are striped horizontally and vertically to convert them into 2D feature maps with spatial dimensions of *L* x *L*, where *L* is the length of the target sequence. This causes such features to occupy twice the number of channels in the input tensor as compared to features defined on residue pairs.

**Feature**	**Feature defined for single residues (1) or residue pairs (2)**	**Dimensionality per residue or residue pair**	**Channels occupied in input tensor**
Sequence profile	1	21	42
MI	2	1	1
MIp	2	1	1
Mean contact potential	2	1	1
PSICOV contact scores	2	1	1
FreeContact (mfDCA) contact scores	2	1	1
CCMpred (plmDCA) contact scores	2	1	1
PSIPRED secondary structure	1	3	6
Shannon entropy in MSA columns	1	1	2
SOLVPRED solvent accessibility	1	1	2
log(1 + sequence separation)	2	1	1
Sequence bounds (channel of ones)	2	1	1
DeepCov covariance matrix	2	441	441
		**Total**	501

Supplementary Table 6 List of dilation rates *d* for each of the 18 residual blocks in the DMPfold ResNet. A dilation rate of *d* = 1 produces regular, non-dilated convolutions.

**Residual block**	1	2	3	4	5	6	7	8	9	10	11	12	13	14	15	16	17	18
**Dilation rate *d***	1	2	1	4	1	8	1	16	1	32	1	64	1	128	1	1	1	1
